# Treatment of Bone and Joint Tuberculosis in France: A Multicentre Retrospective Study

**DOI:** 10.3390/jcm9082529

**Published:** 2020-08-05

**Authors:** Aurélie Guillouzouic, Claire Andrejak, Olivia Peuchant, Geneviève Hery-Arnaud, Farida Hamdad, Philippe Lanotte, Benjamin Gaborit, Louis Bernard, Pascale Bémer

**Affiliations:** 1 Department of Bacteriology, Nantes University Hospital, 1 Place Alexis Ricordeau, 44095 Nantes, France; pascale.bemer@chu-nantes.fr; 2 Respiratory Department, Amiens University Hospital, 80000 Amiens, France; Andrejak.Claire@chu-amiens.fr; 3 Department of Bacteriology, Bordeaux University Hospital, 33000 Bordeaux, France; olivia.peuchant@chu-bordeaux.fr; 4 Department of Bacteriology, Brest University Hospital, 2 Avenue Foch, 29200 Brest, France; genevieve.hery-arnaud@chu-brest.fr; 5 Department of Bacteriology, Amiens University Hospital, 80000 Amiens, France; Hamdad-daoudi.farida@chu-amiens.fr; 6 Department of Bacteriology, Tours University Hospital, 2 Allee Gaston Pages, 37081 Tours, France; philippe.lanotte@univ-tours.fr; 7 Infectious Disease Department, Nantes University Hospital, 1 Place Alexis Ricordeau, 44095 Nantes, France; benjamin.gaborit@chu-nantes.fr; 8 Infectious Disease Department, Tours University Hospital, 2 Allee Gaston Pages, 37081 Tours, France; l.bernard@chu-tours.fr

**Keywords:** spinal tuberculosis, extraspinal tuberculosis, bone and joint tuberculosis, diagnosis, treatment duration

## Abstract

Background: Nine percent of all cases of tuberculosis are bone and joint tuberculosis (BJTB). BJTB occurs in two main forms: spinal (STB) and extraspinal (ESTB). The aim of this study was to compare STB with ESTB in terms of diagnosis, treatment and outcomes. Methods: We collected demographic, clinical, microbiological, treatment duration and outcome data for patients with BJTB in a retrospective multicentre study over a 17-year period. Results: Of the 116 patients included in the study, 69 (59.5%) had STB and 47 (40.5%) had ESTB. The median age was higher in the ESTB group. There were significantly more foreign-born patients in the STB group. The median time for diagnosis was longer for ESTB (6 months) than STB (4 months) (*p* = 0.017). Magnetic resonance imaging was highly reliable for the diagnosis. Direct examination and histology allowed the diagnosis to be made in more than 80% of cases. The median treatment duration of 12 months, regardless of the type of BJTB, was longer than recommended. A favourable outcome was achieved in 91.9% of cases. Conclusion: The management of BJTB remains challenging. An earlier diagnosis should be more effective, reducing the total duration of treatment and leading to better tolerance.

## 1. Introduction

Although the objective of the World Health Organisation (WHO) is eradication of tuberculosis (TB) globally by 2035, this disease remains a real problem of public health, with more than 6.4 million new cases per year and nearly 1.4 million deaths annually worldwide [1]. In Europe, although the TB notification rates are decreasing, the proportion of extrapulmonary TB is increasing, accounting for 19.3% of all TB cases [2]. Bone and joint TB (BJTB) is the third most common form after the pleural and lymphatic disease, and seems to be increasing [2,3], occurring in 9% of all TB cases in two main forms: spinal TB (STB) and extraspinal TB (ESTB). Diagnosis remains a challenge, in particular for atypical localisation. In addition, neurological complications and sequelae are relatively common.

Several published studies have focused on STB [4,5,6,7]. A recent retrospective register-based study included 282 cases of BJTB from 1994 to 2011 in Denmark [8]. This study showed that STB had a poor prognosis, with significant sequelae and high mortality, even in a country with a low incidence of TB [8].

Studies on ESTB are scarce, and have focused mainly on countries with a high incidence of TB [9,10]. We conducted a retrospective multicentre study over a 17-year period in France by collecting demographic, clinical, microbiological, treatment and outcome data on patients with BJTB. The aim of this study was to compare cases of STB and ESTB in terms of anti-tuberculosis drug-use and treatment tolerance, duration of treatment and outcome 1 year after completion of treatment.

## 2. Materials and Methods

### 2.1. Study Design

This study was designed as a multicentre, retrospective, cohort study of patients aged more than 15 years with BJTB. The study protocol was approved by the ethics review boards in each centre (University of Nantes, centre 1, was the coordinating centre). Patient consent was waived because the study was a retrospective analysis of routinely collected data, and there was no study-related intervention.

### 2.2. Study Population

Patients aged more than 15 years were included from January 1997 to December 2013 from five French hospitals. During the study period, the mycobacterial database of each centre was retrospectively analysed to identify patients with a microbiologically confirmed diagnosis of BJTB.

Patients were divided into two groups: (1) STB involving the intervertebral disc and the adjacent vertebrae, with at least one vertebral sample positive for culture of *Mycobacterium tuberculosis* complex (MTC); and (2) ESTB involving at least one bone and/or joint sample positive for culture of MTC without spinal involvement. The patients were excluded if they presented with meningitis associated with BJTB (as the recommended treatment duration varies from 9 to 12 months [11]) or with ESTB- and STB-associated forms.

### 2.3. Data Collection

The available hospital records were reviewed for demographic data, comorbidities (immunosuppression, diabetes mellitus, chronic renal failure), human immunodeficiency virus (HIV) status, clinical symptoms (fever, pain, neurological deficit), BJTB localisation, concomitant TB sites, and imaging findings such as bone erosion, lysis, abscess and spinal cord compression. Paper and electronic records were accessed with appropriate permission.

Paraclinical results, including histological analysis, acid-fast bacilli (AFB) smear results, median time of positive cultures in liquid and solid media, MTC species identification and antibiotic susceptibility were collected. Data were anonymised and recorded in Microsoft Excel 2010 (Microsoft, Redmond, WA, USA).

The diagnostic time between the onset of clinical signs and the first positive culture for MTC was calculated. The mean diagnostic time for ESTB forms was compared with that observed for STB forms.

Details of the drug regimens used for treatment were collected. Median duration of treatment, and its evolution over time, were analysed.

Patients were then classified into 3 groups corresponding to a treatment duration of (1) 6 to 9 months according to the WHO guidelines (group 1), (2) 10 to 12 months according to the median duration of our study (group 2), and (3) more than 12 months for the longest treatment times in our study (group 3). The following characteristics were analysed in the 3 groups of patients: comorbidities, clinical forms, imaging findings, treatment modifications, resistance to anti-tuberculosis drugs, associated surgical treatment, duration of treatment for each centre, and outcome 1 year after the end of treatment. The outcome was considered favourable if the clinical and imaging findings were compatible with cure, and as unfavourable if the clinical or radiological findings were consistent with active TB, and/or if at least one bone and joint sample was positive for culture of MTC.

### 2.4. Microbiological Methods

For a limited number of patients, direct detection of the MTC genome was performed by nucleic acid amplification testing (NAAT) using standardised kits (three different tests were used, the Cobas Amplicor MTB (Roche Diagnostic) test, the Genotype MTBDR (Hain Lifescience, Nehren, Germany) test, the GeneXpert MTB/RIF^®^ (Cepheid) test). For all patients, microscopic examination was performed by Ziehl–Neelsen and/or auramin staining, then specimens were inoculated onto a liquid culture medium (mainly BD BACTEC MGIT 960 system, Becton Dickinson, Le Pont De Claix, France) and a solid culture medium (mainly Löwenstein–Jensen). The identification of MTC at the species level was based on biochemical tests, or more recently on molecular commercial kits (GenoType MTBC; Hain Lifescience, Nehren, Germany). BACTEC 460TB and then MGIT 960 systems were used for testing the susceptibility of MTC strains to rifampicin (R), isoniazid (H), ethambutol (E) and pyrazinamide (Z), which was performed as recommended [12]. In cases of discrepancies or multidrug resistance (MDR) results, the strains were sent to the National Reference Centre for Mycobacteria and anti-tuberculosis drug resistance (Paris, France).

### 2.5. Statistical Analysis

Patient characteristics were described using the median and interquartile range (IQR) for continuous variables and proportions for qualitative variables. Continuous variables were analysed using the Student test or the non-parametric test of Wilcoxon, according to the data distribution, and qualitative variables were determined using the *χ^2^* test or Fisher exact test. Data were explored with univariate analysis.

Statistical significance was achieved when *p* < 0.05, and confidence intervals were calculated at the 95% level. All statistical analyses were done using STATA 12.1 (Stata Corporation, College Station, TX, USA).

## 3. Results

**Figure 1:**
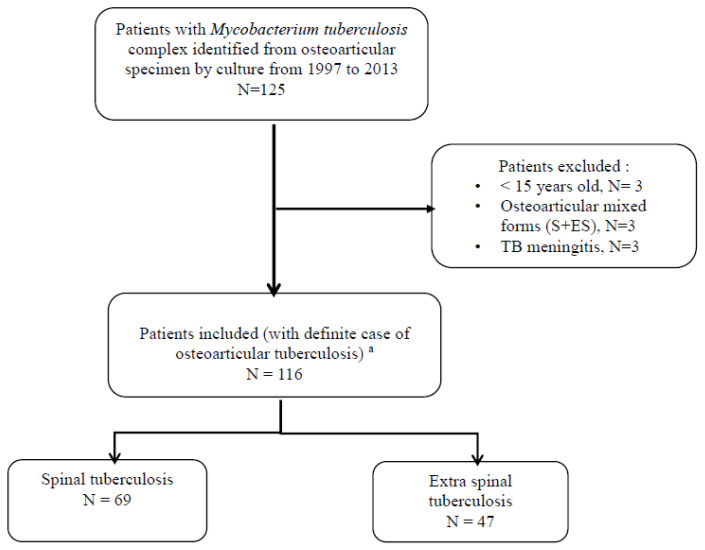
Flowchart for the study. ^a^ Definite cases of bone and joint tuberculosis include patients with Mycobacterium tuberculosis complex, identified from a clinical specimen.

Over the 17-year study period, 116 patients with BJTB were included in the study; 69 (59.5%) with STB and 47 (40.5%) with ESTB (Figure 1). The proportion of extrapulmonary TB among all TB cases was 20%, and the proportion of BJTB among all extrapulmonary TB was 17% in our setting.

### 3.1. Characteristics of the Study Population

The median age was higher in the ESTB group. STB was more frequent among foreign-born patients, most of whom were of African origin. Few comorbidities were found in both groups. More patients with ESTB had had TB previously (*p* = 0.051), and were more immunocompromised (*p* = 0.018) compared to patients with STB (Table 1).

### 3.2. Clinical Findings

**Figure 2:**
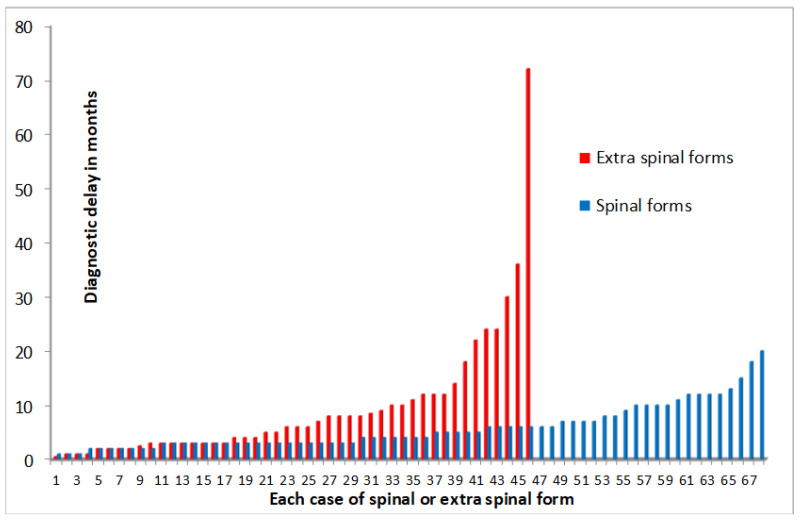
Diagnostic delay according to spinal or extraspinal BJTB forms. ESTB long diagnostic times: 72-month duration in an 80-year-old man with greater trochanter bursitis; 22-month and 24-month durations in elderly patients with a hip and a shoulder prosthesis infection, respectively; 36-month, 30-month and 24-month durations in patients with a rare clinical form.

The median time for diagnosis was significantly longer for ESTB (6 months) than STB (4 months) (*p* = 0.017). Long diagnostic times were observed for six ESTB forms: three in elderly patients and three in patients with a rare localisation (Figure 2).

Pain was the most common symptom. Neurological symptoms were present only in patients with STB (13 of 69, 18.8%) (*p* = 0.002), and sinus tract formation was found only in patients with ESTB (5 of 47, 10.6%) (*p* = 0.006). Concomitant TB sites mainly affected the respiratory tract (28 of 116, 24.1%) (Table 1).

Among those with STB, the lumbar and thoracic spine were most commonly involved (56 of 69, 81.1%). The location of ESTB was varied; half of the cases involved the hip, knee and shoulder (25 of 47, 53.1%) (Table 2).

Abnormal results from computed tomography or magnetic resonance imaging (MRI) were found in 107 of 114 (93.9%) patients (abnormal imaging findings were not observed for 6 patients, and not documented for 1 patient). Erosion or lysis was observed in half of the patients, whatever the group. In the STB group, abscesses were more frequent (*p* < 0.001), and spinal cord compressions were observed (*p* < 0.001) (Table 1). Clinical examination detected a neurological deficit in 18.8% of STB cases, and imaging made it possible to visualise spinal cord compression in 31.9% of cases (Table 1). For patients with ESTB, osteoarthritis, osteitis and bursitis were observed in 34 of 45 (75.5%), 9 of 45 (20.0%) and 8 of 45 (17.8%), respectively.

### 3.3. Paraclinical Results

Histological examination revealed granulomatous lesions, caseous necrosis and/or acid-fast bacilli (AFB) in three-quarters of the patients (Table 3). AFB smears were positive in 38.3% of cases. Cultures tested positive earlier with broth medium compared with solid medium (*p* < 0.001). NAAT for MTC was positive in 21 (72.4%) of the 29 specimens tested, including 9 smears negative for AFB. *M. tuberculosis* was the most commonly identified species (90.5%). Among 116 susceptibility tests results, monoresistance to isoniazid was found in 6 cases (high level, *n* = 4; low level, *n* = 2) and to pyrazinamide in 7 cases (*M. bovis*, *n* = 5; *M. tuberculosis*, *n* = 2). Two strains presented an MDR phenotype: one case of ESTB in a 19-year-old man from Mali, and one case of STB in a 32-year-old man from Gabon.

### 3.4. Treatment

#### 3.4.1. Type of Treatment

The drug regimen was available in 112 cases (refused treatment, *n* = 1; died before the start of treatment, *n* = 1; data unknown, *n* = 2). Patients with a non-MDR strain were treated with the four-drug regimen HRZE (73 of 110, 66.4%) or the three-drug regimen (HRZ or HRE, 37 of 110, 33.6%). The two patients with MDR TB received specifically recommended treatments for 24 months [11].

Fluoroquinolones were added to the anti-tuberculosis treatment in 22 cases of BJTB, 10 cases of ESTB and 12 cases of STB. The additional use of fluoroquinolones was discerned for 20 patients: (1) intolerance to anti-tuberculosis drug (*n* = 8); (2) MDR strains (*n* = 2), isoniazid mono-resistant strains (*n* = 4) and pyrazinamide intrinsically resistant *M. bovis* strains (*n* = 2); (3) unfavourable evolution consistent with active TB (*n* = 3); and (4) localisation of epididymitis (*n* = 1).

Surgery using debridement, pus drainage, laminectomy or synovectomy was required in 25 of the 68 (36.8%) patients with STB, and 13 of the 47 (27.7%) patients with ESTB (*p* = 0.266).

#### 3.4.2. Duration of Treatment

Of the 116 patients, 7 died during the first 2 months, data were unavailable for 8, and 101 were treated for at least 6 months. The median duration of treatment was 12 months for both STB and ESTB groups (Table 1).

#### 3.4.3. Patient Characteristics According to Treatment Duration

The characteristics of the patients according to duration of treatment are shown in Table 4. The duration of treatment was 6 to 9 months for 13 patients, 10 to 12 months for 64 patients (10 and 11 months for 3 patients, 12 months for 61 patients), and more than 12 months for 24 patients (median duration of 18 months, longest duration of 24 months). In groups 2 and 3, treated for 12 months and more, no patients were immunocompromised, and paravertebral abscesses and MCT-resistant strains (isoniazid, *n* = 6; pyrazinamide, *n* = 7) were more common.

Among the 24 patients in group 3 the following results were significant: 5 patients were immunocompromised (malignant hemopathy, *n* = 2; long-term corticosteroid therapy, *n* = 1; kidney transplant patient, *n* = 1; connectivity, *n* = 1); anti-tuberculosis treatment required adaptation in 11 cases (50%, *p* = 0.015) (ethambutol-related optic neuritis, *n* = 2; drug-induced hepatitis, *n* = 2; generalised erythematous rash, *n* = 2; sinus tract formation under treatment, *n* = 2; dose increase due to low plasma drug concentrations, *n* = 1; unknown data, *n* = 2); two MDR *M. tuberculosis* strains required longer treatment duration; 1 patient with associated epididymitis had persistent pain after 15 months of treatment. The outcome was favourable at 1 year in 66.7% of patients after 6 months of treatment, 97.5% after 12 months of treatment, and in 95.5% after more than 12 months of treatment. The outcome was unfavourable for three patients in group 1, and one patient in group 3 (for details, see the legend for Table 4).

The unfavourable outcomes were as follows: group 1 (*n* = 3)—one failure after 9 months of treatment for TB in the knee in an 80-year-old patient with chronic lymphocytic leukaemia; one failure after 8 months of treatment for a TB in the knee in an 85-year-old patient with associated pulmonary and lymph node forms; one case of STB with intolerance to rifampicin requiring the addition of fluoroquinolone. Group 3 (*n* = 1)—sinus tract formation after 9 months of treatment for TB in the hip in an 83-year-old patient, with a favourable outcome after 22 months of treatment. TB non-related death: group 1, *n* = 1; group 2, *n* = 1.

**Figure 3:**
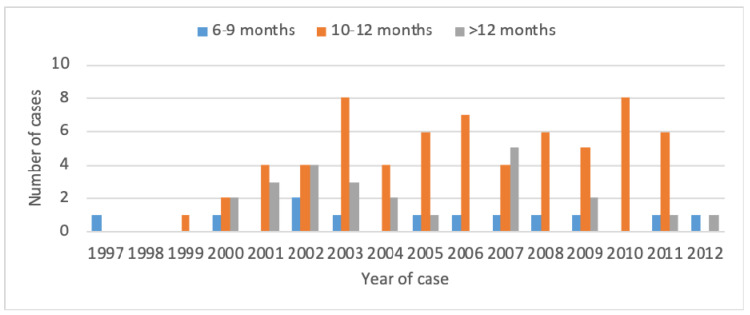
The distribution of patients with regard to duration of treatment for the study period.

The distribution of patients in the three groups was homogeneous during the study period (Figure 3).

## 4. Discussion

The purpose of the current study was to evaluate the management of French patients with BJTB over a 17-year period, based on a collaboration between microbiologists and clinicians.

During the study period, 44% of all cases BJTB occurred in foreign-born patients, in contrast with a higher proportion of foreign-born patients reported in the Netherlands (61%) and Denmark (83%) [3,8]. Furthermore, immigrants accounted for 52.2% of cases of STB, which is lower than is reported in the United Kingdom (92%) and Denmark (85.6%) [5,8], and higher than is reported in Spain, where 33% of cases of STB occur in foreign-born patients [7].

The HIV epidemic has contributed to an increased number of cases of TB worldwide, but no coinfection was found in our cohort, as previously reported [6,8]. Immunosuppressive therapy was reported more frequently in native-born patients with ESTB, as a result of neoplasia, hemopathy, systemic disease or transplant.

The time to diagnosis was significantly longer for ESTB—up to 6 years in our study—due to non-specific clinical presentation, especially in areas with a low prevalence of ESTB, estimated at 1% to 2% [13]. As found in our series, arthritis was more common than osteomyelitis, and bursitis was the most rarely described form [13,14]. Long diagnostic times may occur for elderly or immunocompromised patients, and in cases of rare TB forms in the small bones of the hand and feet [13]. MRI should be performed because plain films may be negative or contribute very little. Erosion and/or lysis in association with arthritis were major imaging signs of ESTB, detected in about half of the cases in our study.

The spine is usually the most common site of BJTB, accounting for 59.5% in our cohort and 65% in a Danish cohort [8], but only 26% in the United Kingdom [15].

A median diagnostic time of 4 months for STB is concordant with the time needed for a spinal lesion to be detected by imaging [16]. A median duration of symptoms of 4 months, before diagnosis of STB, was reported [6]. Pain was the most common symptom, with neurological deficit highly variable in between 23% and 76% of all cases, and a higher prevalence of cervical and thoracic forms [4]. MRI allows the visualisation of cord compression by pus and debris, intrinsic cord signals, bone marrow changes and disc destruction. In our study, 31.9% of cases of spinal cord compression were detected by MRI, in contrast to 18.8% based on clinical examination findings alone, and abscesses were found in 66% of cases of STB. Our results confirm the reliability of MRI, considered to be the most sensitive and specific method for the radiological diagnosis of BJTB [13,16,17].

Almost 40% of our cases were smear positive, similar to results from Denmark, allowing a diagnostic orientation in almost half of the cases [8]. Histological analysis revealed granulomatous disease in more than two thirds of the cases, as reported for extrapulmonary specimens [18]. Histology and direct examination allowed a diagnosis to be made in more than 80% of cases, long before the results of the culture were available.

The median time to the identification of a positive liquid culture in our study was 17 days, and of the patients tested with NAAT, 72.4% tested positive, including cases with AFB smear-negative specimens. The median turnaround time was 2 days. These patients waited 3 weeks less, from the time of biopsy to starting treatment, than those whose samples were not tested. The WHO recommends Xpert MTB/RIF^®^ (Cepheid) for testing samples in patients suspected of having BJTB [19]. Against culture, sensitivities as high as 96% for STB and 100% for BJTB have been reported [20,21,22]. Thus, NAAT in addition to AFB smear, histology and culture should be performed on specimens collected with high suspicion of BJTB [18].

Surgical treatment was required for almost one third of the patients, as expected, with two main indications: drainage of abscesses and laminectomy for neurological decompression [4,8].

In 1986, short-course chemotherapy of 9 months was found to be equivalent to a conventional 18- to 25-month treatment in 350 patients [23]. In 2003 and again in 2016, the Centres for Disease Control and Prevention recommended a standard 6- to 9-month course of rifampin-containing chemotherapy for BJTB [11,24]. The median time for anti-tuberculosis treatment in our study was 12 months, with no difference between STB and ESTB forms, similar to that found in the United Kingdom and Spain, but in contrast to what was described in Denmark during the same period (Table 5) [5,7,8]. The results of our study corroborate the results of an anonymous audit conducted in 2004 among 66 physicians in France [25]. The reported treatment duration of BJTB was 12 months for 64.6% of physicians, and 18 months for 16.9%, revealing a high rate of unnecessarily prolonged treatment for most cases of extrapulmonary TB [25].

We looked for possible reasons that led to these long treatments of up to more than 9 months in our study. Patients treated for between 10 and 12 months were not more immunocompromised. MRI findings showed more abscesses than in the 6- to 9-month treatment group. However, persisting inflammatory changes showing up on follow-up MRI scans, after completing therapy and despite clinical improvement, do not constitute an indication for further treatment [5]. Another explanation of the extended duration of treatment could be the strains resistant to anti-tuberculosis drugs. Again, treatment of no more than 9 months was recommended for isoniazid-resistant tuberculosis (whatever the level of resistance) and pyrazinamide intrinsically resistant *M. bovis* strains [11,26,27].

Intolerances requiring treatment adjustments, which were more frequent in group 3, were not more frequent in the 10- to 12-month group than in the 6- to 9-month group. In the group treated over 18 months, an MDR strain was isolated in two cases, and sinus tract formation under treatment was observed in two patients.

The proportion of overall favourable outcome was 91.9% in our study, in accordance with other European studies [7,8]. The outcomes seemed more favourable in the patients treated for at least 12 months, but this was due to the large number of patients included in groups 2 and 3 (and the low number of patients in group 1). This is the main limitation of our retrospective study. With the exception of MDR TB, the outcome could have been just as favourable after 9 months of treatment. It is important to highlight that the 3 cases of ESTB that failed after 9 months of treatment involved very old and fragile patients with a long history of hip or knee infection.

## 5. Limitation Section

Some important elements are missing in this retrospective multicentre study, such as the detailed treatments during the intensive and continuation phases, the contribution of the IGRA test to the diagnosis of BJTB, and the precise type and number of collected samples.

## 6. Conclusions

The management of BJTB remains a real challenge. However, rapid diagnostic methods now make it possible to begin appropriate treatment quickly. Recommended treatment times should be respected as much as possible. A large prospective randomised trial on the duration of BJTB treatment in areas with a high incidence of TB would be helpful.

## Figures and Tables

**Table 1:** Characteristics of patients with bone and joint tuberculosis.

	STB (*N* = 69), *n*/*N* (%)	ESTB (*N* = 47), *n*/*N* (%)	*p* Value
Male sex	31/69 (44.9)	26/47 (55.3)	0.272
Age (years), median (IQR)	58 (31–76)	71 (36–80)	0.157
15–24 years	6 (8.7)	3 (6.4)	
25–44 years	19 (27.5)	10 (21.3)	
45–64 years	13 (18.8)	8 (17.0)	
≥65 years	31 (44.9)	26 (55.3)	
Foreign-born residents	36/67 (52.2)	15/47 (31.9)	0.031
Africa	26/67 (38.8)	12/47 (25.5)	
Asia	5 (7.5)	1/47 (21.1)	
Others	3/67 (4.5)	2/47 (4.3)	
HIV positive	0/59 (0.0)	0/42 (0.0)	ND
Previous tuberculosis	4/69 (5.8)	8/47 (17.0)	0.051
Comorbidities			
Immunosuppression ^a^	4/65 (6.2)	9/42 (21.4)	0.018
Diabetes mellitus	3/65 (4.6)	3/42 (7.1)	0.579
Chronic renal failure	2/65 (3.1)	2/42 (4.8)	0.654
Clinical features			
Pain	65/69 (94.2)	43/47 (91.5)	0.571
Fever	18/69 (26.1)	15/47 (31.9)	0.495
Impaired general condition ^b^	31/69 (44.9)	16/47 (34.0)	0.241
Neurological deficit	13/69 (18.8)	0/47 (0.0)	0.002
Sinus tract formation	0/69 (0.0)	5/47 (10.6)	0.006
Concomitant tuberculous site	20/66 (30.3)	14/44 (31.8)	0.866
Pulmonary	18/66 (27.3)	10/44 (22.7)	0.592
Extrapulmonary ^c^	2/66 (3.0)	7/44 (15.9)	0.016
Pulmonary and extrapulmonary ^c^	0/66 (0.0)	3/44 (6.8)	0.031
Imaging findings			
Erosion and/or lysis	35/69 (50.7)	25/45 (55.6)	0.614
Abscesses	46/69 (66.7)	8/45 (17.8)	<0.001
Spinal cord compression	22/69 (31.9)	0/45 (0.0)	<0.001
Time to diagnosis (months), median (IQR)	4 (3–7)	6 (3–11)	0.017
Duration of treatment (months), median (IQR)	12 (12–12)	12 (12–12)	0.468

^a^ Immunosuppression, mainly because of essentially systemic corticosteroids, immunosuppressive drugs or cancer. ^b^ Including asthenia, weight loss. ^c^ Other than bone and joint tuberculosis. *p* values < 0.05 are statistically significant. ESTB, extraspinal tuberculosis; IQR, interquartile range; STB, spinal tuberculosis. *n*/*N*: number of cases /total number. ND: not determined.

**Table 2:** Sites of bone and joint tuberculosis.

Site	*n*/*N* (%)
Spinal tuberculosis	69 (59.5)
Lumbar vertebrae	34/69 (49.3)
Thoracic vertebrae	22/69 (31.9)
Thoracolumbar vertebrae	7/69 (10.1)
Cervical vertebrae	6/69 (8.7)
Other bone and joint tuberculosis	47 (40.5)
Hip	10/47 (21.3)
Knee	9/47 (19.1)
Shoulder	6/47 (12.8)
Ankle	5/47 (10.6)
Sacroiliac	4/47 (8.5)
Elbow	4/47 (8.5)
Wrist	3/47 (6.4)
Ribs	2/47 (4.3)
Arm/foot	3/47 (2.1)
Multifocal	3/47 (6.4)

*n*/*N*: number of cases/total number.

**Table 3:** Laboratory results for bone and joint tuberculosis.

	STB and ESTB (*N* = 116)
C reactive protein (mg/L), median (IQR)	38 (20–91)
Histological examination contributing to the diagnosis (*n*/*N*, %)	55/77 (71.4)
Cultures	
Number of specimens analysed, median (IQR)	1 (1–2)
Liquid medium positive delay (days), median (IQR)	17 (11–23)
Solid medium positive delay (days), median (IQR)	26 (20–30)
AFB positive smear (*n*/*N*, %)	41/107 (38.3)
Positive NAAT on sample (*n*/*N*, %)	21/29 (72.4)
Identified species (*n*/*N*, %)	
*M. tuberculosis*	105/116 (90.5)
*M. bovis*	5/116 (4.3)
*M. africanum*	5/116 (4.3)
*M. canettii*	1/116 (0.9)
Anti-tuberculosis resistance (*n*/*N*, %)	
Isoniazid monoresistance	6/116 (5.2)
Pyrazinamide monoresistance	7/116 (6.0)
Multidrug resistance	2/116 (1.7)

STB, spinal tuberculosis; ESTB, extraspinal tuberculosis; *n*/*N*: number of cases /total number. IQR, interquartile range; AFB, acid-fast bacilli; NAAT, nucleic acid amplification testing.

**Table 4:** Characteristics of 101 patients according to the duration of treatment.

	Group 1: 6–9 Months of Treatment (*n* = 13), *n*/*N* (%)	Group 2: 10–12 Months of Treatment (*n* = 64), *n*/*N* (%)	Group 3: Treatment for More Than 12 Months (*n* = 24), *n*/*N* (%)	*p* Value
Predisposing factors				
Previous TB	1/13 (7.7)	8/64 (12.5)	3/24 (12.5)	0.882
Immunosuppression	3/13 (23.1)	3/58 (5.2)	5/22 (22.7)	0.038
Concomitant pulmonary TB	5/13 (38.5)	13/63 (20.6)	8/21 (38.1)	0.175
Extraspinal forms	6/13 (46.1)	26/64 (40.6)	12/24 (50.0)	0.717
Imaging findings				
Erosion/lysis	7/13 (53.8)	34/64 (53.1)	11/23(47.8)	0.900
Abscesses	3/13 (23.1)	33/64 (51.6)	10/23 (43.5)	0.165
Spinal cord compression	3/13 (23.1)	13/64 (20.3)	2/23 (8.7)	0.405
Treatment changes	4/12 (33.3)	10/56 (17.9)	11/22 (50.0)	0.015
Monoresistance to isoniazid	0/13 (0.0)	4/64 (6.3) ^a^	2 ^a^ /24 (8.3)	0.583
Monoresistance to pyrazinamide	0/13 (0.0)	5/64 (7.8) ^b^	2/24 (8.3) ^b^	0.572
Multidrug resistance	0/13 (0.0)	0/64 (0.0)	2/24 (8.3)	0.038
Associated surgical treatment	5/13 (38.5)	22/64 (34.4)	7/24 (29.2)	0.833
Favourable outcome at 1 year	8/12 (66.7) ^c^	39/40 (97.5) ^d^	21/22 (95.5) ^e^	0.009

^a^ High-level resistance, *n* = 4; low-level resistance, *n* = 2. ^b^ *M. bovis*, *n* = 5; *M. tuberculosis*, *n* = 2. ^c^ Patient lost to follow-up, *n* = 1. ^d^ Patient lost to follow-up, *n* = 24. ^e^ Patient lost to follow-up, *n* = 2. *p* values < 0.05 are statistically significant. *n*/*N*: number of cases /total number.

**Table 5:** Recommended and documented duration of treatment for BJTB.

Source	Recommended Treatment Duration (Months)		Median Duration of Treatment (Months)	
	STB	ESTB	STB	ESTB
ATS/CDC/IDSA [11,24]	6–9 ^a^			
World Health Organisation [26,27]	6–9 ^b^			
Dutt [23]			9	
Kenyon [5]			11.6	
Peghin [7]			12.1	
Johansen [8]			9	7
Our study			12	

^a^ Even for pyrazinamide intrinsically resistant *M. bovis* strains. ^b^ Even for isoniazid monoresistant strains, at low or high level. ESTB, extraspinal tuberculosis; STB, spinal tuberculosis. ATS: American Thoracic Society/CDC: Center for Disease Control/IDSA: Infectious Disease Society of America.

## References

[1] World Health Organization (2019). Global Tuberculosis Report 2019.

[2] Sandgren A., Hollo V., van der Werf M.J. (2013). Extrapulmonary tuberculosis in the European Union and European Economic Area, 2002 to 2011. Eurosurveillance.

[3] Jutte P.C., van Loenhout-Rooyackers J.H., Borgdorff M.W., van Horn J.R. (2004). Increase of bone and joint tuberculosis in The Netherlands. J. Bone Jt. Surg. British.

[4] Dunn R.N., Ben Husien M. (2018). Spinal tuberculosis. Bone Jt. J..

[5] Kenyon P.C., Chapman A.L.N. (2009). Tuberculous vertebral osteomyelitis: Findings of a 10-year review of experience in a UK centre. J. Infect..

[6] Pertuiset E., Beaudreuil J., Liote F., Horusitzky A., Kuntz D. (1999). Spinal tuberculosis in adults. A study of 103 cases in a developed country, 1980–1994. Medicine (Baltimore).

[7] Peghin M., Rodríguez-Pardo D., Sanchez-Montalva A., Pellisé F., Rivas A., Tortola T., Aguilar-Company J., Almirante B., Pigrau C. (2017). The changing epidemiology of spinal tuberculosis: The influence of international immigration in Catalonia, 1993–2014. Epidemiol. Infect..

[8] Johansen I.S., Nielsen S.L., Hove M., Kehrer M., Shakar S., Wøyen A.V.T., Andersen P.H., Bjerrum S., Wejse C., Andersen A.B. (2015). Characteristics and clinical outcome of bone and joint tuberculosis from 1994 to 2011: A retrospective register-based study in Denmark. Clin. Infect. Dis..

[9] Ali R., Jalil A., Qureschi A. (2012). Extra spinal osteoarticular tuberculosis: A case series of 66 patients from a tertiary care hospital in Karachi. J. Pak. Med. Assoc..

[10] Chen S.-T., Zhao L.-P., Dong W.-J., Gu Y.-T., Li Y.-X., Dong L.-L., Ma Y.-F., Qin S.-B., Huang H. (2015). The clinical features and bacteriological characterizations of bone and joint tuberculosis in China. Sci. Rep..

[11] American Thoracic Society, CDC, Infectious Diseases Society of America (2003). Treatment of tuberculosis. MMWR Recomm. Rep..

[12] Woods G.L., Brown-Elliott B.A., Conville P.S., Desmond E.P., Hall G.S., Lin G., Pfyffer G.E., Ridderhof J.C., Siddiqi S.H., Wallace R.J. (2011). Susceptibility Testing of Mycobacteria, Nocardiae, and Other Aerobic Actinomycetes.

[13] De Backer A.I., Mortelé K.J., Vanhoenacker F., Parizel P.M. (2006). Imaging of extraspinal musculoskeletal tuberculosis. Eur. J. Radiol..

[14] 14. Leonard M.K., Blumberg H.M. (2017). Musculoskeletal Tuberculosis. Microbiol. Spectr..

[15] Broderick C., Hopkins S., Mack D.J.F., Aston W., Pollock R., Skinner J.A., Warren S. (2018). Delays in the diagnosis and treatment of bone and joint tuberculosis in the United Kingdom. Bone Joint J..

[16] Jain A.K., Sreenivasan R., Saini N.S., Kumar S., Jain S., Dhammi I.K. (2012). Magnetic resonance evaluation of tubercular lesion in spine. Int. Orthop..

[17] Kamara E., Mehta S., Brust J.C.M., Jain A.K. (2012). Effect of delayed diagnosis on severity of Pott’s disease. Int. Orthop..

[18] Lewinsohn D.M., Leonard M.K., LoBue P.A., Cohn D.L., Daley C.L., Desmond E., Keane J., Lewinsohn D.A., Loeffler A.M., Mazurek G.H. (2017). Official American Thoracic Society/Infectious Diseases Society of America/Centers for Disease Control and Prevention clinical practice guidelines: Diagnosis of tuberculosis in adults and children. Clin. Infect. Dis..

[19] World Health Organization (2014). Xpert MTB/RIF Implementation Manual. Technical and Operational ‘how-to’ Practical Considerations.

[20] Held M., Laubscher M., Zar H.J., Dunn R.N. (2014). GeneXpert polymerase chain reaction for spinal tuberculosis: An accurate and rapid diagnostic test. Bone Jt. J..

[21] Gu Y., Wang G., Dong W., Li Y., Ma Y., Shang Y., Qin S., Huang H. (2015). Xpert MTB/RIF and GenoType MTBDR*plus* assays for the rapid diagnosis of bone and joint tuberculosis. Int. J. Infect. Dis..

[22] Shen Y., Yu G., Zhong F., Kong X. (2019). Diagnostic accuracy of the Xpert MTB/RIF assay for bone and joint tuberculosis: A meta-analysis. PLoS ONE..

[23] Dutt A.K., Moers D., Stead W.W. (1986). Short-course chemotherapy for extrapulmonary tuberculosis. Ann. Intern. Med..

[24] Nahid P., Dorman S.E., Alipanah N., Barry P.M., Brozek J.L., Cattamanchi A., Chaisson L.H., Chaisson R.E., Daley C.L., Grzemska M. (2016). Official American Thoracic Society/Centers for Disease Control and Prevention/Infectious Diseases Society of America clinical practice guidelines: Treatment of drug-susceptible tuberculosis. Clin. Infect. Dis..

[25] Tattevin P., Chapplain J.-M., Lesprit P., Billy C., Cazenave-Roblot F., Alfandari S., Bernard L., Rouveix E., Bouvet E. (2006). Tuberculosis treatment duration in France: From guidelines to daily practice. Eur. J. Intern. Med..

[26] World Health Organization (2006). Guidelines for the Programmatic Management of Drug-Resistant Tuberculosis.

[27] World Health Organization (2018). Treatment Guidelines for Isoniazid-Resistant Tuberculosis. Supplement to the WHO Treatment Guidelines for Drug-Resistant Tuberculosis.

